# Clinical Evaluation of Hydrophilic and Hydrophobic Resin-Based Sealants in Uncooperative Children: A 24-Month Randomized Controlled Trial

**DOI:** 10.3390/children13040565

**Published:** 2026-04-18

**Authors:** Hussein A. Alharthy, Amani A. Al Tuwirqi, Alaa A. Nadhrin, Ala A. Aljubour, Layla N. Khogeer, Heba M. Elkhodary

**Affiliations:** 1 Dentistry Department, East Jeddah Hospital, Jeddah First Health Cluster, Jeddah 22233, Saudi Arabia; 2 Pediatric Dentistry Department, Faculty of Dentistry, King Abdulaziz University, Jeddah 22233, Saudi Arabia; anadhreen@kau.edu.sa; 3 Department of Dental Public Health, Faculty of Dentistry, Oral & Craniofacial Sciences, King’s College London, London WC2R 2LS, UK; 4 Dentistry Department, Ad Diriyah Hospital, Riyadh Third Health Cluster, Riyadh 11564, Saudi Arabia; aaljubour@moh.gov.sa; 5 Pediatric Dentistry Department, University Dental Hospital, King Abdulaziz University, Jeddah 22233, Saudi Arabia; lkhogeer@kau.edu.sa; 6 Department of Pedodontics and Oral Health, Faculty of Dental Medicine for Girls, Al Azhar University, Cairo 11651, Egypt

**Keywords:** hydrophilic, hydrophobic, sealant, uncooperative children, retention

## Abstract

**Highlights:**

**What are the main findings?**

- Hydrophilic and hydrophobic resin-based sealants showed comparable retention and cariostatic performance in uncooperative children over 24 months.
- Sealant retention declined significantly over time in both groups, with child behavior emerging as a key factor influencing clinical outcomes.

**What is the implication of the main finding?**

- Moisture-tolerant hydrophilic sealants do not provide a long-term clinical advantage over conventional hydrophobic sealants in uncooperative pediatric patients.
- Behavioral assessment and management should be prioritized when planning pit and fissure sealant therapy in children.

**Abstract:**

Background/Objectives: Dental caries continues to represent a major oral health concern in children, particularly in uncooperative patients, where effective sealant placement is often compromised. This study evaluated the long-term clinical performance of hydrophilic (UltraSeal XT hydro) and hydrophobic (Helioseal-F) resin-based sealants in uncooperative children aged 6–9 years, assessing retention and caries incidence over 24 months. Methods: In a split-mouth, double-blinded randomized controlled trial, 34 children (104 first permanent molars) were enrolled, with 31 participants (98 teeth) completing the study. Sealants were randomly assigned to hydrophilic or hydrophobic group, with assessments at 3, 6, 12, 18, and 24 months. Results: Complete retention declined progressively in both groups, from 59.2% to 2.0% in the hydrophilic group and from 42.9% to 0% in the hydrophobic group at 24 months, with no significant intergroup differences (*p* = 0.719). Caries-free rates decreased from 81.6% to 49.0% in the hydrophilic group and from 75.5% to 40.8% in the hydrophobic group (*p* = 0.293). Children with definitely negative behavior showed significantly lower retention at 6 and 12 months (*p* = 0.006 and *p* < 0.001) compared to those with negative behavior, although differences were not significant at 24 months. Conclusions: Overall, both sealants demonstrated comparable retention and cariostatic performance, indicating that material properties alone do not determine long-term success. Further research should focus on long-term follow-up and comparative evaluation of hydrophilic sealants in cooperative and uncooperative populations to better understand how patient behavior affects sealant performance.

## 1. Introduction

Dental caries in children remains a significant public health burden [1]. A recent report by the World Health Organization (WHO) estimated that nearly 3.5 billion individuals globally are affected by dental caries [2]. In Saudi Arabia, recent statistics show that the prevalence of untreated dental caries in children aged 6–12 years has reached an alarming 96%, according to the Saudi Ministry of Health [3]. Current clinical practice recommends the use of pit and fissure sealants in molars with deep anatomical grooves, which favor biofilm formation and food retention because of their morphology [4].

Pit and fissure sealants have long been recognized as effective in preventing occlusal caries in both primary and permanent molars. A 2008 review by the American Dental Association supports the application of these sealants to prevent occlusal caries, in addition to their role in inhibiting the progression of early non-cavitated carious lesions [5]. Despite their proven benefits, the use of pit and fissure sealants in pediatric patients can be challenging due to children’s behavioral patterns and difficulty in cooperating during the procedure. Achieving adequate moisture control in such patients is often difficult, leading to poor-quality sealant placement with compromised retention. It is hypothesized that using a moisture sealant, especially in children with negative behavior, would result in superior retention and higher longevity. These factors are considered key markers for the success of sealant survival [6]. Conventional resin-based sealants (RBSs) are hydrophobic materials that bond to enamel via micromechanical retention but are highly sensitive to moisture contamination, which may compromise retention and interfere with fissure maturation in newly erupted molars [7,8]. To address these limitations, moisture-tolerant hydrophilic RBSs have been developed. These sealants improve penetration into pits and fissures under less-than-ideal isolation conditions, exhibit enhanced mechanical properties due to higher filler content, and demonstrate bioactive potential through ion exchange with enamel [7,9].

Recent research has increasingly compared hydrophilic and hydrophobic sealants to determine their durability in different clinical settings [10,11]. Notably, Thetsanasalee et al. (2024) [12] evaluated the retention rates of both sealant types in a split-mouth randomized controlled trial carried out in field conditions, demonstrating promising outcomes for hydrophilic materials. However, their study involved cooperative children in community-based settings, where behavior and moisture control were more manageable. To our knowledge, no study conducted in Saudi Arabia has investigated the performance of these sealants in uncooperative children, a population in which moisture control is exceptionally challenging and clinical outcomes are often compromised. This gap underscores the need to assess how these materials perform under such demanding clinical conditions. This randomized controlled trial evaluated, over 24 months, the clinical efficacy of different resin-based pit and fissure sealants, specifically hydrophobic versus hydrophilic types, assessing their retention rates and cariostatic effects in children aged six to nine years.

## 2. Materials and Methods

### 2.1. Study Design

This study was a split-mouth randomized controlled clinical trial conducted over 24 months, with evaluations conducted at 3, 6, 12, 18, and 24 months. The present investigation represents the 24-month follow-up of a previously published 12-month randomized controlled clinical trial [13] involving the same cohort of uncooperative children. The same inclusion criteria, clinical protocol, and evaluation methods were maintained to ensure consistency and enable longitudinal comparison. Ethical approval was obtained from the Committee of Research Scientific Units, Faculty of Dentistry, King Abdulaziz University, Jeddah, Saudi Arabia (Ref. No. 29-04-2020). The study complied with CONSORT guidelines [14] to ensure methodological integrity.

### 2.2. Participants

A total of 34 uncooperative children between 6 and 9 years of age were recruited using a systematic sampling method from the patient list at the Pediatric Dental Clinics of King Abdulaziz University in Jeddah, Saudi Arabia. Parental informed consent was obtained for all participants. Children were included if they were healthy, aged 6–9 years, and had bilaterally erupted maxillary and/or mandibular first permanent molars with deep fissures classified with a score of 0 according to ICDAS II [15]. ICDAS II criteria were used at baseline to confirm that included molars were caries-free and eligible for sealant placement. Subsequent longitudinal evaluations of sealant retention and caries outcomes were performed using the CCC Sealant Evaluation System. Only uncooperative children who were rated as “definitely negative” characterized by clear refusal of treatment, intense crying, marked fear, or other evident signs of extreme negative behavior, or as “negative” indicating reluctance to accept treatment, uncooperative behavior, and a mildly negative attitude such as being sullen or withdrawn, though not strongly resistant, according to the Frankl Behavior Scale [16], were eligible for inclusion in the study. Uncooperative behavior in the present study was primarily related to dental anxiety, fear, young age, and limited tolerance for dental procedures rather than physical or intellectual disabilities. Children were excluded if they had developmental dental defects, missing or restored contralateral molars, poor oral hygiene, allergies to resin or latex, or systemic disorders.

### 2.3. Sample Size

Sample size estimation was performed based on research by Khatri et al. (2015) [17], which indicated that 64 teeth were required to ensure 80% statistical power at a significance level of 0.05. To account for potential attrition over the 24-month follow-up period, a total of 104 teeth were included. Block randomization was employed to achieve balanced distribution of the materials used for treatment between the two sides. Each block number with their corresponding treatment sequences, as specified in the randomization table, was placed in sealed, non-transparent envelopes. The allocation list was securely stored by an independent member of the dental staff to keep the operator blinded to the allocation. The Bilateral mandibular and/or maxillary first permanent molars were assigned randomly to two groups: Group I (study group), 52 molars sealed with hydrophilic resin-based sealant (UltraSeal XT^®^ hydro™, Ultradent Products, South Jordan, UT, USA); Group II (control group), 52 molars sealed with hydrophobic resin-based sealant (Helioseal-F, Ivoclar-Vivadent, Amherst, NY, USA).

### 2.4. Data Collection

Baseline data, including medical history, age, sex, and nationality, were recorded before the intervention. Prior to sealant application, the medical history of each child was reviewed, and baseline data were recorded. Sealant application followed the manufacturer’s instructions, including pre-procedural oral hygiene and dietary instructions. The procedure was performed by a single calibrated operator using non-pharmacological behavior management techniques to encourage child cooperation. Before sealant application, prophylaxis was done. Prophylaxis was performed using a rotary brush with non-fluoridated pumice to remove plaque and debris from the occlusal surface. The fissures were cleaned using an air–water spray without additional mechanical instrumentation. Enamel etching was carried out for 15–20 s according to the manufacturer’s instructions, followed by rinsing for approximately 10 s and gentle air drying. After sealant placement and light curing, each sealant was clinically inspected to ensure complete coverage and to detect bubbles or occlusal interference, and adjustments were made as needed. Then, the occlusal surface was thoroughly rinsed and air-dried with air–water spray. During the application, teeth were isolated using a saliva ejector and cotton rolls. Etching of the enamel surfaces was performed using 35% phosphoric acid (Ultradent Products, USA) for Group I and 37% phosphoric acid etchant (Total Etch, Ivoclar Vivadent, Schaan, Liechtenstein) for Group II, followed by rinsing and drying. Sealants were applied in line with the manufacturer’s guidelines and light-cured for a duration of 20 s using a standardized curing device (Elipar™, Curing Light 2500, 3M EPSE, St. Paul, MN, USA). Clinical follow-up evaluations were carried out at 3, 6, 12, 18, and 24 months post application. Two trained and calibrated evaluators, blinded to group assignments, assessed sealant retention and caries status using the Color, Coverage, and Caries scoring system [18]. Sealant retention was categorized as follows: A (sealant present on all fissure systems), B (sealant present on more than 50% of fissure patterns), C (sealant present on less than 50% of fissure patterns), and D (sealant absent). Caries coding were defined as follows: 0, surface sound, no caries; 1, initial enamel caries subdivided to (1W: white spot lesion; 1B: brown spot lesion); 2, enamel caries; 3P, caries into dentin cavity < 0.5 mm; 3L, caries into dentin cavity > 0.5 mm; and 4, caries with probable pulpal involvement. Behavior classification was recorded at baseline for eligibility and subgroup analysis and was not formally reassessed at each follow-up visit.

### 2.5. Statistical Analysis

Data collection and analysis were performed using the Statistical Package for the Social Sciences (SPSS, version 25). Descriptive statistics characterized the sample, and within-group comparisons of sealant retention and caries occurrence after 3, 6, 12, 18, and 24 months were u using Friedman’s test. Intergroup comparisons used the Mann–Whitney U test after 3, 6, 12, 18 and 24 months, with a significance level set at α = 0.05.

## 3. Results

### 3.1. Study Population and Baseline Characteristics

**Figure 1:**
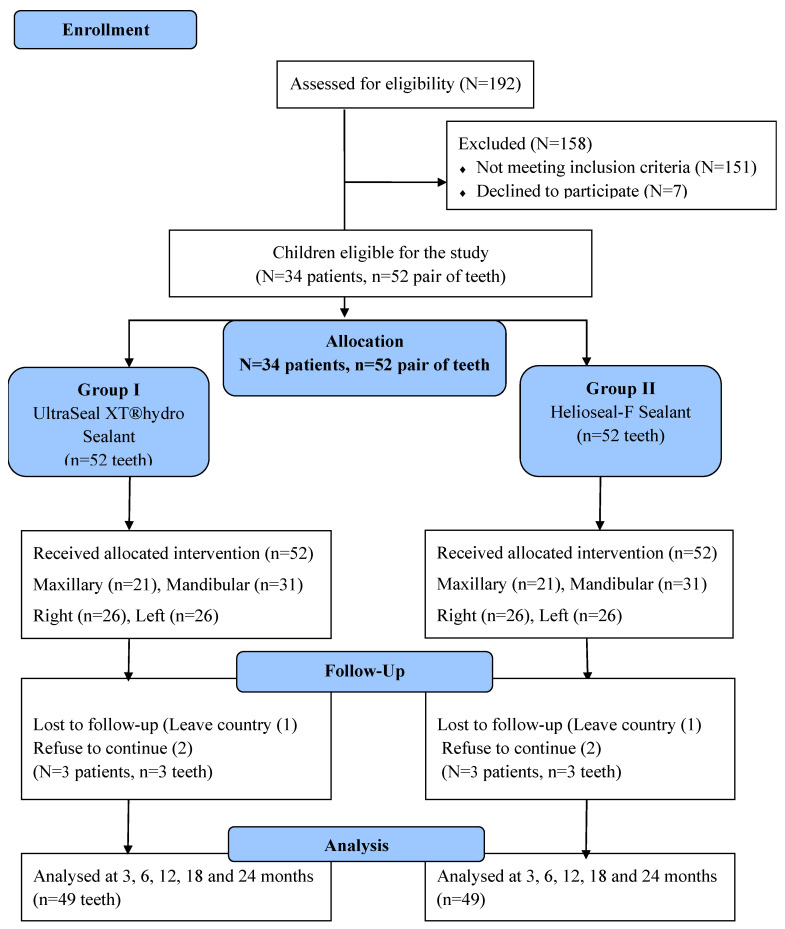
Flow diagram of the study through the 24-month follow-up period.

In total, 104 molars from 34 uncooperative children were enrolled and evaluated over 24-month period, with follow-up assessments at 3, 6, 12, 18, and 24 months as shown in the study flow diagram (Figure 1). After enrollment, three children dropped out before the first follow-up visit, resulting in a final analysis of 31 participants with 98 teeth (49 pairs). The children had a mean age of 7.46 ± 1.14 years, ranging from 6 to 9.9 years. The study population included 57.7% males and 42.3% females. Most children were classified as having “negative” behavior (76.5%), followed by “definitely negative” behavior (23.5%).

### 3.2. Sealant Retention

Table 1 presents the differences in sealant retention between the study groups across all follow-up periods. In both groups, sealant retention gradually declined over time. The proportion of teeth with complete sealant retention (Score A) in Group I (hydrophilic sealant) decreased from 59.2% at 3 months to 2.0% at 24 months. In Group II (hydrophobic sealant), complete retention declined from 42.9% at 3 months to 0% at 24 months. At the final follow-up, complete sealant loss (Score D) was observed in 42.9% of Group I and 34.7% of Group II. Sealant retention decreased significantly over time in both groups (*p* < 0.001). Nevertheless, there were no statistically significant differences between the groups at any follow-up point, including the 24-month evaluation (*p* = 0.719). These findings were consistent with the 12-month results, where sealant retention similarly declined with no significant intergroup differences (*p* = 0.707), indicating a sustained reduction in retention over time for both materials.

### 3.3. Caries Incidence

Table 2 presents the differences in caries scores between the study groups across all follow-up periods. Both groups showed a progressive increase in caries incidence over the follow-up period. At 12 months, 81.6% of teeth in Group I and 75.5% in Group II remained caries-free. By 24 months, these values declined to 49.0% and 40.8%, respectively. Enamel caries (Score 2) was found in 12.2% of Group I and 20.4% of Group II at 24 months. However, no statistically significant difference in caries incidence was observed between the two groups at any follow-up interval, including at 24 months (*p* = 0.293), consistent with the 12-month findings (*p* = 0.447).

### 3.4. Sealant Retention and Caries Outcomes According to Child Behavior

Table 3 and Table 4 summarize the differences in sealant retention and caries scores, respectively, between children exhibiting negative and definitely negative behavior across all follow-up periods. When sealant performance was analyzed according to child behavior, retention decreased over time in both behavior categories (negative and definitely negative). At 6 and 12 months, children with definitely negative behavior demonstrated significantly lower sealant retention compared with those exhibiting negative behavior (*p* = 0.006 and *p* < 0.001, respectively). Although the same trend continued at the 24-month follow-up, the difference between the groups was not statistically significant (*p* = 0.078). Regarding caries outcomes, no statistically significant difference was observed between the two behavior categories at 24 months (*p* = 0.293). The highest percentages of enamel caries (Score 2) in both negative and definitely negative behavior groups were recorded at the 24-month follow-up (16.7% and 15.4%, respectively). At the end of the study period, 37.5% of children in the negative behavior group and 65.4% in the definitely negative behavior group were caries-free. The 24-month results showed no significant differences between the two behavior categories in terms of sealant retention or cariostatic effect, which aligns with the overall trend observed during the study.

## 4. Discussion

This split-mouth, double-blind randomized controlled trial evaluated the retention and cariostatic effects of hydrophilic and hydrophobic RBS in uncooperative children. Several important findings were revealed. Both sealant types demonstrated a progressive decline in retention over time, with complete retention (Score A) decreasing markedly from baseline to the 24-month follow-up in both groups. No statistically significant differences were found between the hydrophilic (UltraSeal XT^®^ hydro™) and hydrophobic (Helioseal-F) sealants regarding retention rates or caries prevention during the entire follow-up period. Child behavior had a significant impact on clinical outcomes at the 6- and 12-month follow-ups; however, this effect was not statistically significant at 24 months. This 24-month study builds upon our previous 12-month randomized controlled trial conducted on the same cohort of uncooperative children [13]. The previous study reported a comparable trend, showing a gradual reduction in sealant retention and no statistically significant differences between hydrophilic and hydrophobic sealants regarding retention or caries prevention. The present long-term evaluation confirms and extends those findings, suggesting that both materials exhibit comparable performance even over an extended follow-up period.

In pediatric dentistry, placing RBS can be particularly challenging due to difficulties achieving adequate isolation, ensuring patient cooperation, and maintaining proper moisture control. These challenges are especially evident in uncooperative children, children with special health care needs, very young patients, and those with excessive salivation or partially erupted teeth. The inconsistent findings reported in previous studies, along with the limited evidence regarding the retention of hydrophilic RBS, highlight the need for further investigation. Therefore, conducting this study was essential to evaluate and compare the caries prevention effectiveness of hydrophobic and hydrophilic RBS under conditions of compromised moisture control, such as those encountered in uncooperative pediatric patients [13].

To date, clinical trials evaluating the effectiveness of hydrophilic RBS in uncooperative children are lacking, which justified the inclusion of this population in the present study. The 24-month evaluation period was selected because it represents a clinically relevant follow-up interval for assessing the medium-term retention and caries prevention effectiveness of RBS. Previous clinical studies have demonstrated that most sealant failures occur within the first two years after placement [4,5]; therefore, a 24-month follow-up provides evidence regarding the materials’ durability and clinical performance. The age group of 6–9 years was selected as it coincides with the eruption stage of the first permanent molars, a period when these teeth are particularly vulnerable to caries [19]. Both upper and lower molars were included, as previous studies have shown no significant differences in sealant effectiveness between arches [20]. The split-mouth design allowed direct comparison of both materials within the same patient, thereby minimizing the influence of between-subject factors such as diet, oral hygiene, and caries risk. Selection bias was minimized through randomization, and the double-blind design involving both participants and evaluators reduced the risk of assessment bias.

Caries detection was performed using visual and tactile methods in accordance with the ICDAS II criteria, avoiding the use of sharp explorers, which are no longer recommended for caries detection due to the risk of enamel damage [7]. Partial isolation using cotton rolls and high- and low-volume suction was employed, as rubber dam placement is often challenging in uncooperative children and partially erupted teeth and may require local anesthesia, thereby limiting its clinical feasibility in such cases [21]. The degree of moisture control and drying feasibility was not quantitatively stratified among participants, which may have influenced sealant retention outcomes. Previous evidence suggests that partial isolation can achieve acceptable sealant retention without compromising clinical outcomes [21]. The experimental sealant, UltraSeal XT^®^ hydro™, was selected for its moisture-tolerant formulation and high filler content, which have been suggested to enhance bonding and retention [9]. Helioseal-F was chosen as the control material due to its well-documented clinical performance, fluoride release, and stable filler composition. The 24-month follow-up period exceeded the critical early failure window of sealant retention, allowing for meaningful assessment of long-term outcomes [9].

The observed decline in sealant retention over the 24 months aligns with existing literature on sealant longevity in pediatric populations. The present study represents the 24-month follow-up of our previously published 12-month randomized controlled trial on the same cohort of uncooperative children [10]. The one-year evaluation showed comparable retention and cariostatic performance between hydrophilic and hydrophobic sealants, with early reduction in retention already evident. Extending the follow-up to 24 months allowed assessment of medium-term performance beyond the initial adaptation phase. The current findings confirmed the earlier results, demonstrating a continued decline in retention without significant differences between materials, suggesting that long-term outcomes are influenced more by clinical and behavioral factors than by sealant hydrophilicity alone. Bhat et al. (2013) [8], Askarizadeh et al. (2017) [22], and Priyadharshini et al. (2021) [23] reported comparable findings, showing no significant differences between hydrophilic and hydrophobic RBS. These findings are consistent with those of a recent systematic review and meta-analysis conducted by our team [24], which found no significant difference in retention between hydrophilic and hydrophobic RBS, which reported no significant difference in retention between hydrophilic and hydrophobic RBS, as well as with the 12-month findings of Thetsanasalee et al. (2024) [12]. Although the marked reduction in complete retention at 24 months (2.0% in the hydrophilic group and 0% in the hydrophobic group) is concerning, it is not unexpected given the challenges of moisture control and patient cooperation in this population. Similar conclusions were reported by Garrocho-Rangel et al. (2023) [6], who identified moisture contamination as a major factor compromising sealant retention in pediatric patients. Some first permanent molars were partially erupted at the time of sealant placement, which may have further complicated moisture control and contributed to reduced retention. In the present study, maxillary and mandibular first molars showed no clinically relevant arch related differences in sealant retention or caries outcomes at 24 months. However, previous studies have reported inconsistent findings regarding arch effects, suggesting that anatomical features, occlusal loading, and operator access may all influence RBS performance [8,12,13,25,26].

Caries incidence increased progressively in both groups over time, with caries-free rates declining from over 75% at 12 months to approximately 45% at 24 months. This finding is consistent with previous reports, including the study by Prabakar et al. (2018) [26]. Thetsanasalee et al. (2024) similarly reported comparable retention performance between hydrophilic and hydrophobic RBS under field conditions, with no significant differences in caries prevention [12]. The absence of statistically significant differences in caries incidence between the sealant types in the present study suggests that both materials provide comparable cariostatic effects when adequately retained. This result reinforces the concept that the clinical effectiveness of RBS is primarily determined by sustained material retention and integrity rather than by hydrophilicity alone [5].

The absence of a significant difference between hydrophilic and hydrophobic RBS challenges the theoretical advantage that hydrophilic materials are expected to have in the moisture-contaminated oral environment. This finding suggests that the clinical benefits of moisture-tolerant formulations may be less pronounced than anticipated, particularly in the long term. Several factors may explain this observation. First, the degree of moisture contamination during application may not have been severe enough to demonstrate the advantages of hydrophilic materials. Second, both materials may be equally susceptible to the mechanical stresses imposed by occlusal forces over time.

Child behavior influenced RBS performance during the early follow-up periods. Children with definitely negative behavior exhibited significantly lower sealant retention at 6 and 12 months compared to those with negative behavior, indicating that patient cooperation affects short-term outcomes. At the 24-month follow-up, the differences in retention were no longer statistically significant, indicating that behavioral factors may mainly influence early sealant survival rather than long-term outcomes. Similarly, caries incidence at 24 months did not differ significantly between the two behavior categories, with 37.5% of children in the negative behavior group and 65.4% in the definitely negative group remaining caries-free. These findings suggest that while behavioral assessment can help anticipate early sealant failure, sealant material properties and long-term outcomes are not solely determined by patient behavior.

This study has several strengths, including its randomized, double-blinded, split-mouth design, which minimized bias and controlled for confounding variables. To our knowledge, this study represents the first randomized controlled trial designed to compare hydrophilic and hydrophobic RBS in uncooperative children. Additionally, calibration of the operator and evaluators enhanced the reliability and validity of the results.

The results of this study should be interpreted in light of several limitations. First, the study included only uncooperative children, which may restrict the generalizability of the findings to the wider pediatric population. Second, the study design did not include a cooperative control group, which would have provided valuable context for understanding the magnitude of behavioral impact on sealant performance. Third, the 24-month follow-up period, while substantial, may not capture the full long-term performance differences between materials that might emerge over extended periods. Lack of detailed caries risk factors like biofilm control, dietary sugar intake, fluoride exposure, or behavioral and socioeconomic status represent another limitation in this study. In addition, child behavior was assessed at baseline for eligibility and subgroup analysis but was not longitudinally reassessed at each follow-up visit, which may limit interpretation of behavioral changes over time. Longer-term studies would provide more definitive evidence regarding material durability and caries prevention efficacy. Local anesthesia was not routinely required for fissure sealing and was referenced only in relation to potential rubber dam placement challenges in uncooperative children. An alternative clinical approach for uncooperative children may involve the use of temporary glass ionomer sealants as an interim strategy, given their moisture tolerance. Definitive RBS could then be placed once improved cooperation and adequate moisture control are achieved. Future studies should consider including a glass ionomer control group versus hydrophilic RBS to better evaluate the relative effectiveness of moisture-tolerant sealants in uncooperative pediatric populations.

## 5. Conclusions

Both hydrophilic (UltraSeal XT^®^ hydro™) and hydrophobic (Helioseal-F) Resin-Based Sealant demonstrated comparable retention and cariostatic performance in uncooperative children over 24 months, indicating that sealant material properties alone may not determine long-term clinical success. Child behavior influenced sealant retention during the early follow-up periods, highlighting the value of behavioral assessment in anticipating short-term outcomes. Future research should include longer-term follow-up, and cooperative control groups.

## Figures and Tables

**Table 1:** Comparison of sealant retention between study groups across all follow-up periods.

Groups	Scores	Follow-Up Periods (Months)					*p*-Value †
		3	6	12	18	24	
		n (%)	n (%)	n (%)	n (%)	n (%)	
Group I	A	29 (59.2)	9 (18.4)	2 (4.1)	2 (4.1)	1 (2.0)	<0.001 *
	B	14 (28.6)	32 (65.3)	27 (55.1)	17 (34.7)	9 (18.4)	
	C	5 (10.2)	5 (10.2)	12 (24.5)	19 (38.8)	18 (36.7)	
	D	1 (2.0)	3 (6.1)	8 (16.3)	11 (22.4)	21 (42.9)	
Group II	A	21 (42.9)	7 (14.3)	2 (4.1)	1 (2.0)	0 (0.0)	<0.001 *
	B	24 (49.0)	33 (67.3)	24 (49.0)	21 (42.9)	8 (16.3)	
	C	3 (6.1)	7 (14.3)	16 (32.7)	17 (34.7)	24 (49.0)	
	D	1 (2.0)	2 (4.1)	7 (14.3)	10 (20.4)	17 (34.7)	
*p*-value ∝		0.234	0.638	0.707	0.681	0.719	

n: Number of teeth, Group I: UltraSeal XT^®^ hydro™ sealant, Group II: Helioseal-F Sealant, A: Sealant present on all of the fissure system, B: Sealant present on >50% of fissure pattern but some missing, C: Sealant present on <50% of fissure pattern, D: No sealant present, †: Friedman test, ∝: Mann–Whitney U test, *: Statistically Significant *p* < 0.05.

**Table 2:** Difference in caries scores among study groups at all follow-up periods.

Groups	Scores	Follow-Up Periods (Months)					*p*-Value †
		3	6	12	18	24	
		n (%)	n (%)	n (%)	n (%)	n (%)	
Group I	0	49 (100)	47 (95.9)	40 (81.6)	32 (65.3)	24 (49.0)	<0.001 *
	1	0 (0.0)	2 (4.1)	5 (10.2)	12 (24.5)	19 (38.8)	
	2	0 (0.0)	0 (0.0)	4 (8.2)	5 (10.2)	6 (12.2)	
Group II	0	49 (100)	46 (93.9)	37 (75.5)	30 (61.2)	20 (40.8)	<0.001 *
	1	0 (0.0)	3 (6.1)	6 (12.2)	13 (26.5)	19 (38.8)	
	2	0 (0.0)	0 (0.0)	6 (12.2)	6 (12.2)	10 (20.4)	
*p*-value ∝		1.00	0.651	0.447	0.658	0.293	

n: Number of teeth, Group I: UltraSeal XT^®^ hydro™ sealant, Group II: Helioseal-F Sealant, 0: Surface sound, no caries, 1: Initial enamel caries, 2: Enamel caries, †: Friedman test, ∝: Mann–Whitney U test, *: Statistically Significant *p* < 0.05.

**Table 3:** Difference in sealant retention among negative and definitely negative behavior at all follow-up periods.

Behavior	Scores	Follow-Up Periods (Months)					*p*-Value †
		3	6	12	18	24	
		n (%)	n (%)	n (%)	n (%)	n (%)	
Negative behavior	A	38 (52.8)	15 (20.8)	4 (5.6)	3 (4.2)	1 (1.4)	<0.001 *
	B	29 (40.3)	48 (66.7)	43 (59.7)	32 (44.4)	15 (20.8)	
	C	5 (6.9)	8 (11.1)	18 (25)	26 (36.1)	31 (43.1)	
	D	0 (0.0)	1 (1.4)	7 (9.7)	11 (15.3)	25 (34.7)	
Definitely negative behavior	A	12 (46.2)	1 (3.8)	0 (0.0)	0 (0.0)	0 (0.0)	<0.001 *
	B	9 (34.6)	17 (65.4)	8 (30.8)	6 (23.1)	2 (2.7)	
	C	3 (11.5)	4 (15.4)	10 (38.5)	10 (38.5)	11 (42.3)	
	D	2 (7.7)	4 (15.4)	8 (30.8)	10 (38.5)	13 (50.0)	
*p*-value ∝		0.290	0.006 *	<0.001 *	0.006 *	0.078	

n: Number of teeth, A: Sealant present on all of the fissure system, B: Sealant present on >50% of fissure pattern but some missing, C: Sealant present on <50% of fissure pattern, D: No sealant present, †: Friedman test, ∝: Mann–Whitney U test, *: Statistically Significant *p* < 0.05.

**Table 4:** Comparison of caries scores among study groups at each follow-up period.

Behavior	Scores	Follow-Up Periods (Months)					*p*-Value †
		3	6	12	18	24	
		n (%)	n (%)	n (%)	n (%)	n (%)	
Negative behavior	0	72 (100)	69 (95.8)	55 (76.4)	43 (59.7)	27 (37.5)	<0.001 *
	1	0 (0.0)	3 (4.2)	8 (11.1)	19 (26.4)	33 (45.8)	
	2	0 (0.0)	0 (0.0)	9 (12.5)	10 (13.9)	12 (16.7)	
Definitely negative behavior	0	26 (100)	24 (92.3)	22 (84.6)	19 (73.1)	17 (65.4)	0.037 *
	1	0 (0.0)	2 (7.7)	3 (11.5)	6 (23.1)	5 (19.2)	
	2	0 (0.0)	0 (0.0)	1 (3.8)	1 (3.8)	4 (15.4)	
*p*-value ∝		1.00	0.651	0.447	0.658	0.293	

n: Number of teeth, 0: Surface sound, no caries, 1: Initial enamel caries, 2: Enamel caries, †: Friedman test, ∝: Mann–Whitney U test, *: Statistically Significant *p* < 0.05.

## Data Availability

Data is available upon reasonable request to the authors.
